# Postoperative and Recurrent Hematuria after Pretransplant Core Needle Biopsy in Living Donor Kidney Transplant

**DOI:** 10.1155/2022/5274521

**Published:** 2022-07-28

**Authors:** Yazan Al-Adwan, Navdeep Singh, Pranit N. Chotai, Farjad Siddiqui, Ashley Limkemann, Austin Schenk, Jayanthan Subramanian, W. Kenneth Washburn, Musab Alebrahim, Amer Rajab

**Affiliations:** Division of Transplantation, The Comprehensive Transplant Center, The Ohio State University Wexner Medical Center, Columbus, OH, USA

## Abstract

**Background:**

Core needle and wedge biopsies are the two main pathologic ways to determine the suitability of a kidney allograft and to have a baseline allograft biopsy in case of future rejection. *Case Presentation*. A 57-year-old patient developed a renal arteriovenous fistula causing postoperative and recurrent hematuria after allograft pretransplant renal core needle biopsy and treated with selective Interventional radiology coil embolization.

**Conclusion:**

Delayed profound hematuria can be seen after pretransplant core needle renal biopsies and can recur again even after complete resolution, due to arteriovenous fistula formation in the renal calyceal system.

## 1. Introduction

Core needle renal biopsy is widely performed percutaneously to evaluate and determine the kidney transplant rejection etiologies [1]. Although there is no clear agreement when to perform a pretransplant allograft baseline biopsies at the time of kidney transplants, allograft biopsies are widely performed among transplant centers using either core needle or wedge biopsies even carrying the risk for arteriovenous fistula formation to the renal calyceal system which might be as high as 16% [2–4]. At our transplant center, guidelines protocol is to perform a backtable baseline allograft biopsy for living donor allografts at the time of transplant as for a baseline histological record in case for posttransplant kidney rejection.

AVF after kidney biopsy is a result of mechanical trauma by the biopsy needle causing a communication between the arterioles and the adjacent venules, and it is the most common complication of allograft kidney biopsy. During kidney transplants, in most of the cases where the AVF is formed due to the core needle, biopsy remains asymptomatic and resorbed spontaneously within weeks [4, 5] but might develop hematuria, hypertension, and renal insufficiency. In the majority of kidney transplants, the AVF-related hematuria can be detected immediately after the kidney reperfusion intraoperatively.

AV fistulas have been traditionally identified by angiography; however, US Color-Doppler examination is considered less invasive and nonnephrotoxic [2].

Here, we report a case of postoperative onset profound hematuria and a late recurrent hematuria as a complication of the core needle biopsy was performed on the backtable of a kidney allograft.

## 2. Case Presentation

A 57-year-old male patient with end-stage renal disease due to type-2 DM, who had a living related donor, was listed for kidney transplant at our transplant center. His past medical history included T2DM, obstructive sleep apnea, hypertension, pulmonary embolism in 2017, secondary to bilateral lower limbs deep vein thrombosis (on Eliquis 5 mg twice daily), and overweight with a body mass index of 31.5 kg/m^2^ (height 177 cm, weight 99.7 kg) and preoperative serum creatinine of 6.17 mg/dL.

The patient had his elective living related kidney transplant from a living related donor who was a 56-year-old male with significant medical history of hypertension and body mass index of 31 kg/m^2^ (height 192 cm, weight 114 kg) with a serum creatinine of 0.99 and creatinine clearance of 153. The hand-assisted donor nephrectomy went uneventfully and revealed single renal artery, vein, and ureter. The allograft kidney was taken to the recipient operating room, and on the backtable, a baseline upper pole core needle biopsy was performed using a 16-gauge needle, which resulted in 15 glomeruli with 0 sclerosis.

The kidney implant went smoothly, and the allograft kidney was reperfused with a cold ischemia time of 10 minutes and a warm ischemia time of 25 minutes; the kidney started to make clear urine immediately after the kidney reperfusion. The ureterovesical anastomosis was carried down over a double-J ureteric stent. After completing the operation, the patient was awakened from anesthesia and taken to the postanesthesia care unit (PACU). Clear urine was collecting in the urine bag at this time. 30 minutes after arrival to the PACU, the indwelling Foley's catheter started to show a profound hematuria with clots in the urine collecting tube and bag. The patient remained hemodynamically stable.

At this time, we replaced the Foley catheter with a 3-way urine catheter and immediately started a continuous bladder irrigation (CBI) to prevent clot formation in the urinary system. The patient's vital signs stayed stable. However, his hemoglobin level dropped from 11.3 g/dL to 9.3 g/dL, and the serum creatinine dropped to 5.32 mg/dL from 6.17 mg/dL.

**Figure 1:**
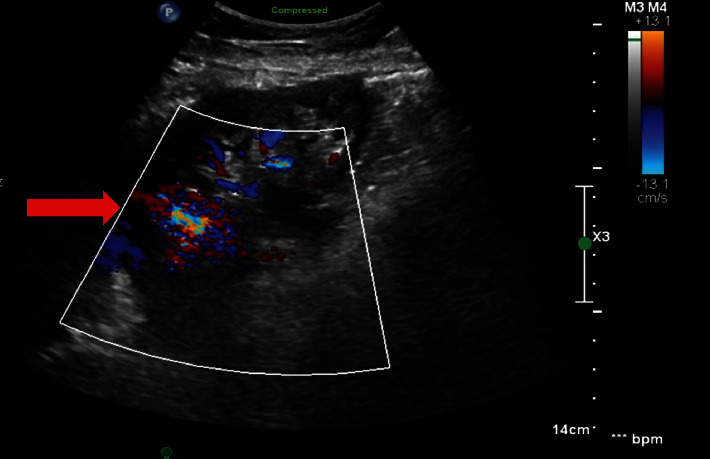
In the superior pole there is a focal area of marked increased flow (arrow) with disorganized echoes cyst suggesting an arteriovenous fistula.

A bedside ultrasound of the transplanted kidney was done, which showed no hydronephrosis and no perinephric fluid collections, but the color-Doppler sonography in the superior pole showed a focal area of marked increased flow with disorganized audible echo cluster suggesting an arteriovenous fistula (Figure 1), giving the history of allograft renal biopsy. The bladder irrigation was continued until the urine cleared again. The patient was taken to the transplant surgical floor fully awake and in a stable condition.

The patient received the appropriate perioperative induction and maintenance immunosuppression regimens as per our transplant center protocols. The patient continued to make an adequate urine output postoperatively, and the renal allograft function showed stable metabolic profile with a trending down serum creatinine.

The CBI was stopped at the third day postoperatively, and the patient was discharged home the following day after the urine cleared normally without having hematuria, and the Foley's catheter was removed, and the patient was voided with no problems, and his Eliquis was resumed as well.

**Figure 2:**
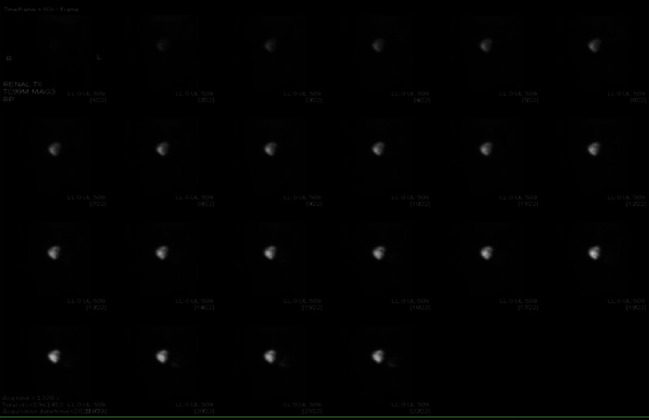
Radionuclide angiogram demonstrates normal blood flow to the right lower quadrant transplant. Subsequent renogram images demonstrate cortical retention of radiopharmaceutical without definite excretion with rising renogram curve, suggestive of ATN.

Once resolved, the hematuria is atypical to recur. Nevertheless, on postoperative day 6, he was readmitted to the hospital complaining of recurrent hematuria, clots passage in urine stream, and urine retention. Foley's catheter was placed; continues bladder irrigation was started. During the hospital stay, adequate hydration was given, Eliquis was stopped and switched with subcutaneous heparin. Urology trial to upsize the Foley's catheter size and continue the bladder irrigation was done with slight improvement over time, but the patient sill passing minimal clots with the hematuria. At this time, we did a radionuclide angiogram scan was done and showed normal blood flow to the transplanted kidney with cortical retention of radiopharmaceutical without definite excretion with rising renogram curve, which is suggestive of acute tubular necrosis (Figure 2).

As a further workup, we decided to do kidney biopsy to rule out the possibility of rejection. The biopsy showed acute tubular necrosis (ATN) with rare small tubular oxalate crystals and no evidence of acute rejection.

The clots kept forming in the bladder causing further decrease in the urine output flow, so the urology team performed cystoscopy, massive clot evacuation, direct bladder irrigation, and stent removal in the operating room. Cystoscopy also revealed tinged blood was dripping from the ureteral orifice at the ureterovesical anastomosis.

**Figure 3:**
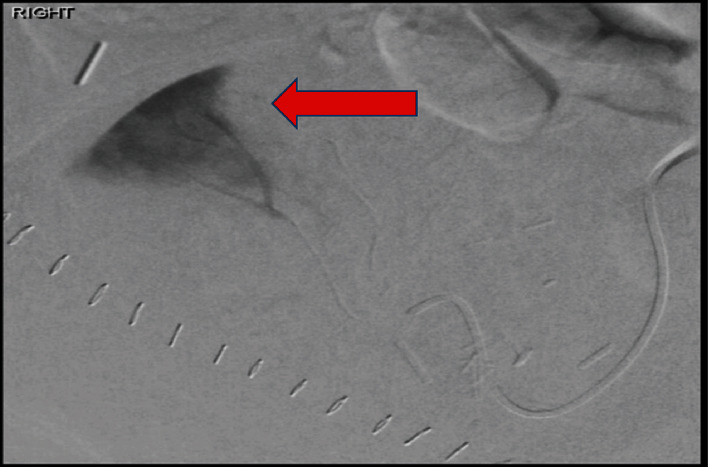
Interpolar region segmental renal artery (arrow): a small focus of early arterial filling was noted with associated early venous drainage demonstrating AVF.

**Figure 4:**
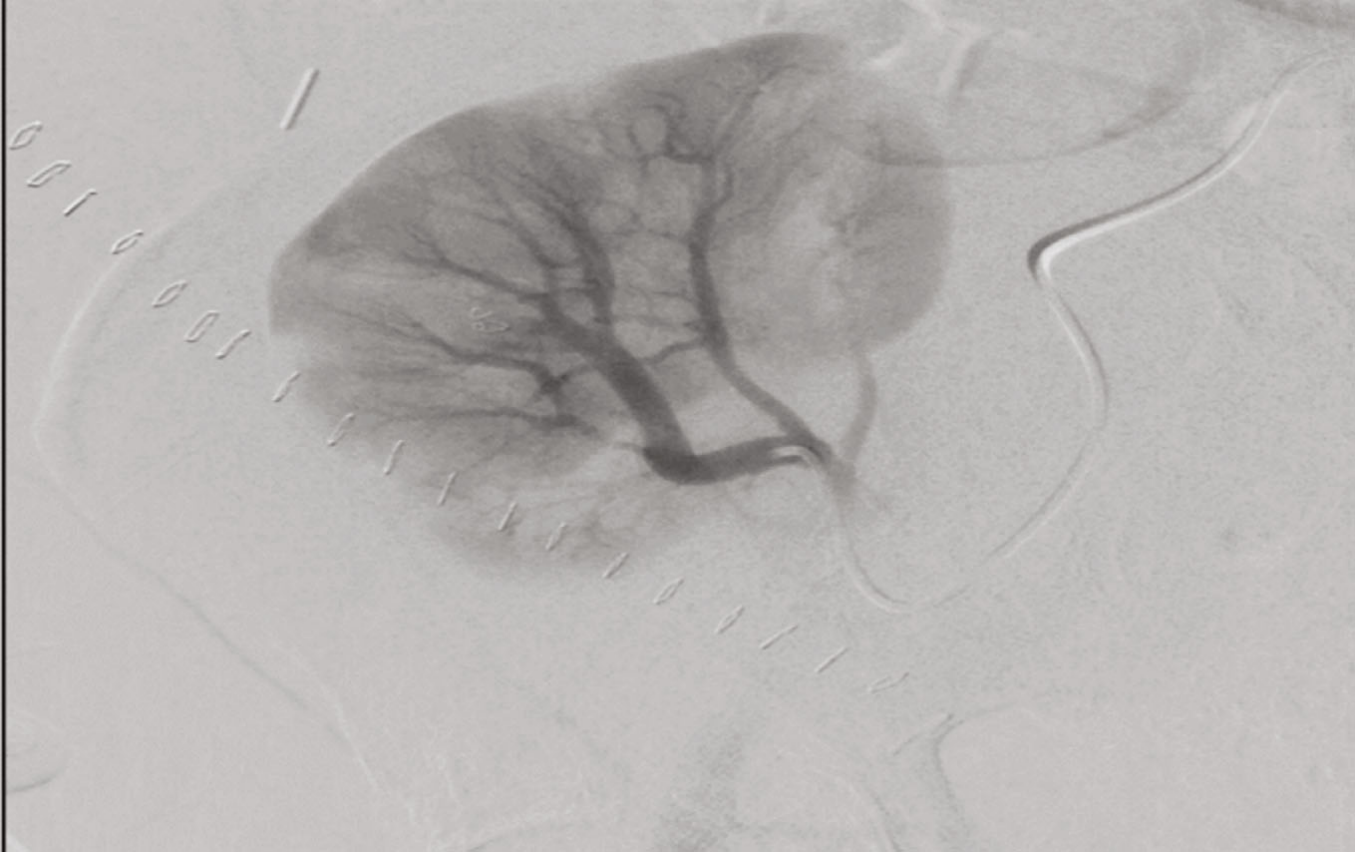
Postembolization arteriogram demonstrates resolution of the venous drainage area.

Although we understand the contrast nephrotoxic effect and the possibility of partial graft function loss might occur with angiography, we decided to take the patient to the IR hoping to control the AVF with the minimum graft function damage using superselective transcatheter embolization [6, 7]. In IR, a segmental interpolar renal artery in the upper pole suggesting the arteriovenous fistula location was successfully selected, embolized, and resolved the fistula (Figures 3 and 4).

Over the next 24-48 hours, CBI was stopped, the hematuria resolved completely, serum creatinine went down, and the patient was discharged home. As of 9 months after this event, his serum creatinine level has plateaued at 1.4 mg/dL.

## 3. Discussion

Kidney transplant is the definitive treatment for end-stage renal disease. The number of patients on the kidney transplant waiting list far outstrips the availability of donated kidneys, and this led to the consideration of the extended criteria donor allografts carrying the high risk of chronic tissue injury. Thus, obtaining a baseline allograft biopsy at time of transplant is crucial to predict graft delayed function and guide therapy, and many transplant centers are routinely performing these biopsies to identify baseline conditions, subclinical rejection, and histological changes under current immunosuppression regimen, drug nephrotoxicity, viral infection, and recurrence of glomerulonephritis [8].

At present, surgeons trend to perform pretransplant allograft biopsies, either a needle biopsy or wedged biopsy. Needle biopsy requires the use of a special punch needle that obtains tissue sample of the allograft organ, while wedge biopsy is performed through surgical excision of a small and adequate part of the allograft organ. There is no clear evidence to prove which technique is superior, regardless of the slight difference in each specific complications; wedge biopsy is superior in obtaining more glomeruli, pathology, and higher Remuzzi score for tissue injury detection [9].

As in transplant centers, protocols, and surgeons' preferences, core needle renal biopsy is widely performed, in terms of a pretransplant biopsy, percutaneous native kidney biopsy—to assess the native kidney disease, and posttransplant percutaneous kidney biopsies.

All these different ways carry the risk for AVF formation as one of the vascular complications of allograft biopsy that may occur with or without bleeding. Allograft pretransplant biopsy-related AVF has an incidence as high as 16.2%, and hematuria can be detected in about 8% [2–4].

The literature showed that posttransplant renal biopsy carries a higher postbiopsy hematuria rate of 9.9% than native renal biopsy of 4.2% [3], as well as an incidence AVF rate of 0.3-6% in native kidney and 10-16% in transplanted kidney biopsies. This wide variation between the two methods and among transplant centers is mainly related to different factors including; the routine use of US during the biopsy procedure, the timing of follow up ultrasound after the biopsy is taken, patient's BMI, the biopsy's operator experience, and the type and size of the used needle during the biopsy procedure.

The majority of AV fistulas have no symptoms and are resorbed spontaneously within weeks. Less than 30% of AV fistulas have clinically manifested with high blood pressure, renal function deterioration, and different degrees of macroscopic hematuria up to such that needs blood transfusion, embolization, or surgery [4]. Hematuria in most cases is detected in the immediate postreperfusion time in the allograft implantation as a macroscopic bleeding through the allograft ureter, and once resolved, it is unlikely to recur.

Many diagnostic methods are used to detect AV fistulas including color-Doppler US, MRI, and angiography, with the latest carrying an advantage of being therapeutic via transcatheter embolization which is indicated in few cases such as heart failure symptoms (shortness of breath, fatigue, and dyspnea or radiological features or congestion), nonspontaneous AVF closure 3 months after the biopsy or persistent gross hematuria, as in our patient. Superselective catheterization permits accurate placement of metallic coils directly into the single artery that typically feeds the AVF, thereby minimizing the vascular territory at risk for infarction [10–12].

In our patient's case, angiographic success (complete occlusion of the vascular lesion) and clinical success (symptom resolution) were achieved.

On the other hand, does the needle size matter?

Studies compared biopsies which were taken using 14-gauge and 16-gauge needles, which showed similar diagnostic accuracy of renal biopsy of native kidneys which is similar for both needle gauges; the potential for complication rates is less using a 16-gauge needle [13–15].

In comparison between 16 and 18 gauge needles, the16-gauge needles provide more glomeruli and more diagnostically adequate renal tissue, with fewer cores without a significant increase in complications compared with 18G needles [14, 16]. Based on these observations, 16-gauge needle was preferably considered by the majority as the first option in kidney biopsies.

In conclusion, in spite of the widespread use of core needle biopsy, as one of the methods to obtain a baseline allograft renal biopsy, it still carries some risk complications including AVF as one of the major vascular complications and it might be devastating as massive bleeding leading to hemorrhagic shock [17]. The effect of the forming AVF can be seen immediately or even in a late fashion, and it might affect the graft function as well.

Transcatheter arterial embolization is effective and safe treating AVF that occurs due to needle biopsy of renal allografts with preservation of most the kidney parenchymal graft.

## Abbreviations

AVF: Arteriovenous fistula
DM: Diabetes mellitus
CBI: Continuous bladder irrigation
ATN: Acute tubular necrosis
IR: Interventional radiology
US: Ultrasound.
